# Association of the C-reactive protein-triglyceride-glucose index (CTI) with lower-extremity peripheral artery disease among U.S. adults

**DOI:** 10.1016/j.athplu.2026.100568

**Published:** 2026-05-19

**Authors:** Sahil Ghay, Baneet Kaur, Agata Piotrowska, Crystal Barroca, Christos G. Mihos, Esteban Escolar, Medeona Gjergjindreaj, Priscilla Wessly, Tarec K. Elajami, Gervasio A. Lamas, Francisco Ujueta

**Affiliations:** a Semyon and Janna Friedman Advanced Research Institute at Mount Sinai Medical Center, Miami Beach, FL, USA; b Division of Cardiology at Mount Sinai Medical Center, Miami Beach, FL, USA; c Department of Internal Medicine, Mount Sinai Medical Center, Miami Beach, FL, USA; d Aurora Cardiovascular and Thoracic Services, Aurora Sinai/Aurora St. Luke's Medical Center, Milwaukee, WI, USA; e New York Medical College, School of Medicine, Valhalla, NY, USA; f Division of Cardiovascular Medicine, Department of Medicine, Vanderbilt University Medical Center, Nashville, TN, USA

**Keywords:** Peripheral artery disease, C-reactive protein-triglyceride-glucose index, Atherosclerosis, Cardiometabolic risk

## Abstract

**Introduction:**

Peripheral artery disease (PAD) is a common yet underrecognized consequence of systemic atherosclerosis, affecting over 200 million adults worldwide and one in five older adults in the United States. PAD carries substantial morbidity, increasing the risk of major cardiovascular and limb events and contributing to declines in quality of life. Inflammation and metabolic dysfunction are key drivers of atherosclerosis. The C-reactive protein-triglyceride-glucose index (CTI) integrates these pathways. We examined whether CTI is associated with PAD in U.S. adults.

**Methods:**

National Health and Nutrition Examination Survey (NHANES) data from 1999 to 2004 were analyzed. Adults aged ≥20 years with available ankle-brachial index (ABI), fasting glucose, triglycerides, and C-reactive protein (CRP) were included. PAD was defined as ABI <0.90; individuals with ABI ≥1.40 were excluded. CTI was calculated as 0.412 × ln(CRP [mg/L]) + ln(triglycerides [mg/dL] × fasting glucose [mg/dL]/2). Logistic regression models evaluated CTI continuously and quartiles, adjusting for demographic, metabolic, and clinical covariates. Multinomial models assessed associations across ABI categories, including borderline ABI (0.90-0.99).

**Results:**

Among 5869 adults, 420 (7%) had PAD. Each 1-unit increase in CTI was associated with higher odds of PAD (aOR 1.47; 95% CI 1.25-1.75; p < 0.001). Higher CTI levels were associated with increased odds of PAD, with the strongest association observed in the highest quartile (aOR 2.07; 95% CI 1.41-3.06; p < 0.001). CTI was also linked to both borderline ABI and clinical PAD in multinomial analyses.

**Conclusion:**

Higher CTI is independently associated with PAD and may provide complementary information for vascular risk evaluation.

## Highlights

• CTI, a composite inflammatory-metabolic index, was independently associated with peripheral artery disease in U.S. adults.
• Each 1-unit increase in CTI was associated with 47% higher adjusted odds of PAD, with a clear dose-response across quartiles.
• The highest CTI quartile had more than twofold higher odds of PAD compared with the lowest quartile, even after adjustment.
• CTI was associated with clinical PAD and borderline ABI, suggesting relevance for early or subclinical disease.
• CTI improved PAD discrimination beyond metabolic risk alone, outperforming the TyG index and enhancing model fit and AUC.

## Graphical abstract

Metabolic dysfunction and inflammatory activation converge to promote endothelial injury, lipid dysregulation, and foam cell formation, accelerating atherosclerotic plaque development and instability within peripheral arteries. This integrated pathophysiologic pathway underlies the development and progression of peripheral artery disease and is captured by CTI. Created in BioRender. Kaur, B. (2026).

**Figure:**
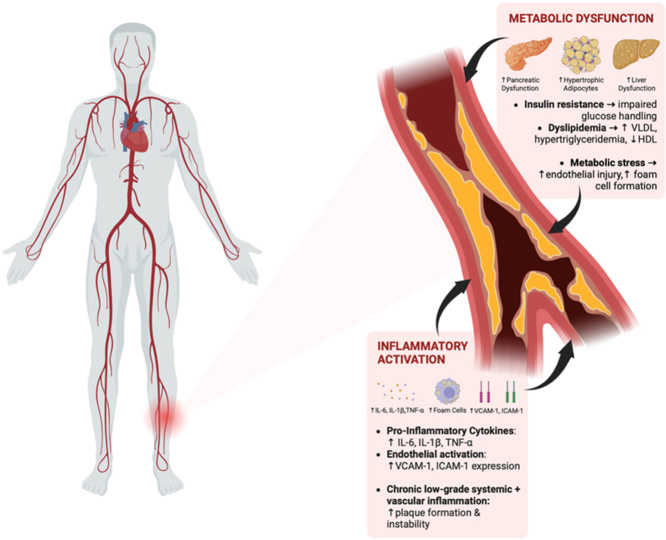

## 1 Introduction

Peripheral artery disease (PAD) is an underrecognized vascular sequela of systemic atherosclerosis affecting more than 200 million individuals worldwide, including 12% to 20% of adults older than 60 years of age in the United States [[1], [2], [3], [4], [5]]. PAD is a highly morbid manifestation of cardiovascular disease (CVD) that heightens risk of major adverse cardiovascular events (MACE), such as myocardial infarction (MI) and stroke, and major adverse limb events (MALE), including tissue necrosis and non-traumatic lower-extremity amputation, as well as impaired quality of life [4,6,7]. Within five years of diagnosis, PAD carries a near 20% rate of MACE and 10-15% mortality rate, with cohort data indicating that up to 75% of patients ultimately pass from a cardiovascular (CV) event [8]. Despite its substantial public health impact and strong association with long-term CV morbidity and mortality, PAD remains underdiagnosed and undertreated, largely due to a significant portion of individuals presenting with asymptomatic or atypical symptoms, leading to delayed detection and missed opportunities for early intervention [1,9].

A higher prevalence of PAD is seen in individuals with predisposing risk factors, including hyperlipidemia, hypertension, diabetes, chronic kidney disease (CKD), and cigarette smoking [[10], [11], [12]]. The presence of three or more risk factors confers nearly a ten-fold increase in disease risk [3,13]. PAD arises from progressive atherosclerotic narrowing and thrombosis of the abdominal aorta, iliac vessels, and most commonly lower-extremity arteries, including aortoiliac, femoropopliteal, and infrapopliteal arterial segments [1,8,14]. As arterial stenosis worsens, initially asymptomatic symptoms progress to exertional symptoms such as intermittent claudication, with further progression of disease leading to chronic limb-threatening ischemia (CLTI) with ischemic rest pain, non-healing ulcers, and tissue necrosis [1,10,15,16]. The pathophysiology of PAD is driven by an interplay of vascular, inflammatory, and metabolic pathways. Plaque formation and instability is perpetuated by endothelial dysfunction, oxidative stress, and chronic inflammation, with further amplification by CV risk factors that upregulate inflammatory mediators including interleukin-6 (IL-6), monocyte chemoattractant protein-1 (MCP-1), vascular cell adhesion molecule-1 (VCAM-1), and reactive oxygen species (ROS) [10,15,16]. Additionally, dyslipidemia and insulin resistance act as key pathophysiologic drivers of PAD, functioning both independently and synergistically to accelerate atherosclerosis within peripheral vessels [17].

The diagnosis of lower-extremity PAD relies on a combination of clinical evaluation and objective vascular testing, with the ankle-brachial index (ABI) serving as the primary screening tool [1,18]. However, ABI has limitations, including falsely normal or non-compressible readings in individuals with a history of renal disease and diabetes secondary to medial arterial calcification [18]. Given these limitations, biomarker-based screening can be an important complementary or alternative approach for timely detection of PAD [18]. The C-reactive protein-triglyceride-glucose index (CTI) is a novel composite biomarker that integrates inflammation, insulin resistance, and dyslipidemia (Graphical Abstract) and has been identified as a strong predictor of all-cause and CV mortality in patients with coronary artery disease and type 2 diabetes [19,20]. To date, however, CTI has not been evaluated as a prognostic marker in individuals with lower-extremity PAD. In this study, we examined the association between CTI and lower-extremity PAD.

## 2 Methods

**Fig. 1:**
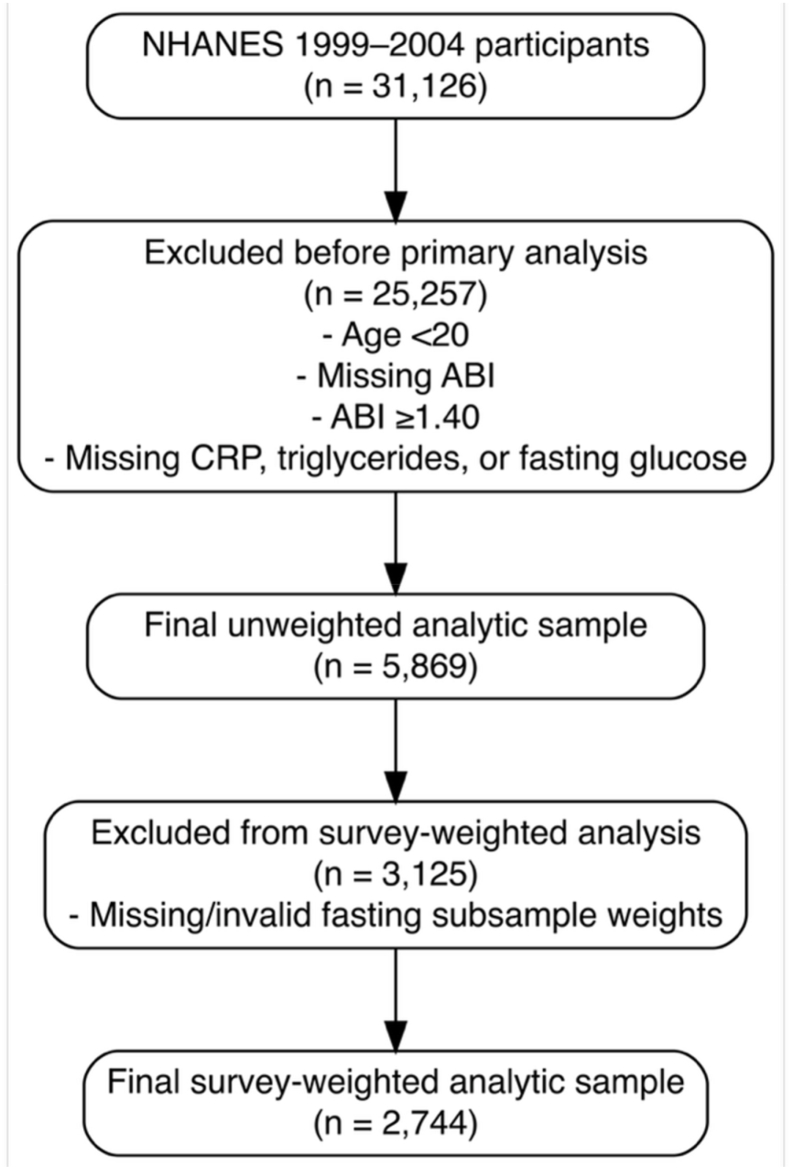
Study population selection flow diagram Flow diagram of cohort selection. From 31,126 participants, exclusions yielded an unweighted sample of 5869. After removing those with missing/invalid fasting weights, 2744 remained for survey-weighted analyses.

In this cross-sectional study, we analyzed data from the National Health and Nutrition Examination Survey (NHANES) from 1999 to 2004. Adults aged 20 years or older with available ABI, fasting triglycerides, fasting glucose, and C-reactive protein (CRP) measurements were included. Participants were excluded if they were younger than 20 years of age, had non-compressible arteries (ABI >1.4), or lacked any of the required laboratory values. Participant selection is detailed in Fig. 1. CTI was calculated using formula 0.412 × ln(CRP [mg/L]) + ln(triglycerides [mg/dL] × fasting glucose [mg/dL]/2) [20]. ABI is derived from Doppler systolic pressures, with values < 0.90 indicating PAD and ≥1.40 suggesting non-compressible arteries, particularly in diabetes and chronic kidney disease [1,21]. PAD was defined as an ABI <0.9 in either leg. [1,21] Because NHANES is fully de-identified and publicly available, no additional Institutional Review Board approval was required [22]. All analyses were performed using R version 4.5.1 (R Foundation for Statistical Computing, Vienna, Austria).

The initial analysis employed a series of logistic regression models to investigate the relationship between CTI and PAD. Three models were constructed. The first model was unadjusted. The second model included established demographic and socioeconomic risk factors for PAD, including age, sex, race and ethnicity, marital status, income-to-poverty ratio, and body mass index. The fully adjusted model contained 16 covariates and incorporated all variables from model two, along with comorbid conditions such as hypertension, coronary heart disease, congestive heart failure, and diabetes. It also included physical activity, smoking status, and laboratory values such as hemoglobin A1c (HbA1c), total cholesterol, high-density lipoprotein cholesterol (HDL), and serum creatinine. Subsequently, we excluded participants with missing ABI measurements, missing CTI laboratory components, or incomplete covariate data. CTI was evaluated continuously as well as by quartiles. CTI was divided into four quartiles with ranges Q1 (6.15-8.54), Q2 (8.55-9.13), Q3 (9.14-9.71), and Q4 (9.72-14.25). In addition, we evaluated a broader set of metabolic and inflammatory biomarkers, including the triglyceride-to-HDL cholesterol ratio, METS-IR, CRP, systemic immune-inflammation index (SII), visceral adiposity index (VAI), and neutrophil-to-lymphocyte ratio (NLR). To enable direct comparison across biomarkers, each marker was standardized, and logistic regression models were used to estimate odds ratios per 1-standard deviation increase in the full cohort. These analyses were used to compare the relative strength of association of each biomarker with PAD.

In addition to the primary analyses, survey-weighted analyses were conducted using design-based logistic regression models to account for the sampling design of NHANES. Fasting subsample weights were applied to obtain nationally representative estimates. Participants with missing survey design variables or invalid or non-positive fasting weights were excluded from the weighted analyses, resulting in a reduction in the analytic sample compared with the unweighted models.

Multinomial logistic regression was then used to evaluate whether higher CTI levels were associated with lower ABI categories across the full ABI spectrum. ABI was categorized into normal (1.00-1.39), borderline (0.90-0.99), and abnormal (<0.90), with the normal group serving as the reference. This approach allowed us to evaluate whether CTI increased not only in overt PAD but also in individuals with borderline ABI who may have early or subclinical vascular disease.

Data were then analyzed using a restricted cubic spline analysis to test whether the relationship between CTI and PAD was linear or nonlinear, using the fully adjusted model. CTI was modeled using four knots placed at the 5th, 35th, 65th, and 95th percentiles. A Wald chi-square test was used to evaluate CTI's overall association with PAD and test for nonlinearity in the spline model. Receiver operating characteristic (ROC) curve analysis was performed to assess the ability of CTI-based models to discriminate PAD. Predicted probabilities from the unadjusted model (model 1), the partially adjusted model (model 2), and the fully adjusted model (model 3) were used to construct ROC curves for PAD. For each model, the area under the curve (AUC) and corresponding 95% confidence intervals were calculated to summarize discriminative performance. To evaluate whether CTI provided incremental discriminatory value beyond metabolic risk alone, parallel ROC analyses were performed using the triglyceride-glucose (TyG) index. Discriminative performance of CTI and TyG-based models was compared using differences in AUC (ΔAUC) and DeLong's test. In addition, model fit was compared between CTI and TyG-based models using differences in Akaike information criterion (ΔAIC). In addition, likelihood ratio tests were performed to compare nested models and evaluate whether CTI provided incremental information beyond a model of all covariates in the fully adjusted model (Model 3), as well as models incorporating comparator biomarkers, including the TyG index, CRP, and the triglyceride-to-HDL cholesterol ratio.

We performed subgroup analyses to assess whether the association between CTI and PAD was consistent across clinically relevant strata. Fully adjusted logistic regression models were repeated within subgroups defined by sex, race, body mass index category, age (<65 vs ≥ 65 years), hypertension, coronary heart disease, and diabetes. For each subgroup, we estimated the odds ratio for PAD per one-unit increase in CTI with corresponding 95% confidence intervals. Likelihood ratio tests comparing models with and without a CTI-by-subgroup interaction term were used to evaluate statistical interaction.

## 3 Results

**Baseline Characteristics:** Among 5869 adults with complete data, 420 had PAD (Table 1). Compared with those without PAD, individuals with PAD were older (70.62 vs 58.68 years, p < 0.001) and had lower income-to-poverty ratios (2.28 vs 2.89, p < 0.001). PAD was more common among non-Hispanic Black adults (23.3% vs 16.8%, p < 0.001) and those with lower educational attainment (42.6% vs 30.6%, p < 0.001). Clinically, the PAD group had higher rates of hypertension (61.9% vs 39.6%, p < 0.001), coronary heart disease (15.5% vs 5.3%, p < 0.001), heart failure (9.3% vs 2.9%, p < 0.001), and stroke (8.8% vs 3.2%, p < 0.001). They also had higher levels of metabolic and inflammatory markers, including fasting glucose (112.98 vs 103.30 mg/dL, p < 0.001), HbA1c (6.03% vs 5.74%, p < 0.001), triglycerides (169.93 vs 158.55 mg/dL, p = 0.002), and CRP (0.64 vs 0.45 mg/L, p < 0.001). Systolic blood pressure (141.61 vs 130.17 mmHg, p < 0.001), serum creatinine (1.03 vs 0.89 mg/dL, p < 0.001), and BUN (17.62 vs 14.49 mg/dL, p < 0.001) were also higher in the PAD group. Other variables, including sex, BMI, HDL, albumin, and hemoglobin, showed no statistically significant differences.

**Table 1:** **Baseline Characteristics of Adults With and Without Peripheral Artery Disease.***Summary of key demographic and clinical characteristics for individuals with and without PAD.*

Variable	No PAD (n = 5449)	PAD (n = 420)	p-value
Age (years)	58.68 (12.62)	70.62 (11.60)	<0.001
Sex			0.504
Male	2807 (51.5)	224 (53.3)	
Female	2642 (48.5)	196 (46.7)	
Race/Ethnicity			<0.001
NH White	3008 (55.2)	237 (56.4)	
NH Black	913 (16.8)	98 (23.3)	
Mexican American	1151 (21.1)	67 (16.0)	
Other Hispanic	213 (3.9)	10 (2.4)	
Other Race	164 (3.0)	8 (1.9)	
Marital Status			<0.001
Not Married	1928 (35.4)	189 (45.0)	
Married	3521 (64.6)	231 (55.0)	
Education Level			<0.001
< High School	1665 (30.6)	178 (42.6)	
High School/GED	1269 (23.3)	105 (25.1)	
Some College	1386 (25.5)	80 (19.1)	
College or Higher	1123 (20.6)	55 (13.2)	
Income-to-Poverty Ratio (PIR)	2.89 (1.61)	2.28 (1.47)	<0.001
Physical Activity			<0.001
Yes	3176 (58.3)	175 (41.7)	
No	2273 (41.7)	245 (58.3)	
Hypertension History			<0.001
Yes	2159 (39.6)	260 (61.9)	
Coronary Heart Disease			<0.001
Yes	290 (5.3)	65 (15.5)	
Heart Failure			<0.001
Yes	158 (2.9)	39 (9.3)	
Stroke			<0.001
Yes	173 (3.2)	37 (8.8)	
Cancer History			<0.001
Yes	617 (11.3)	74 (17.6)	
BMI (kg/m^2^)	28.45 (5.50)	27.91 (5.51)	0.070
Waist Circumference (cm)	98.94 (13.79)	100.23 (13.01)	0.028
Systolic BP (mmHg)	130.17 (20.33)	141.61 (24.02)	<0.001
Diastolic BP (mmHg)	72.81 (13.87)	65.66 (17.62)	<0.001
Hemoglobin A1c (%)	5.74 (1.11)	6.03 (1.16)	<0.001
Glucose (mg/dL)	103.30 (37.70)	112.98 (45.79)	<0.001
Total Cholesterol (mg/dL)	209.63 (41.29)	206.40 (46.35)	0.030
HDL (mg/dL)	52.85 (16.29)	51.12 (15.65)	0.020
Triglycerides (mg/dL)	158.55 (148.66)	169.93 (155.47)	0.002
CRP (mg/L)	0.45 (0.72)	0.64 (1.14)	<0.001
Serum Creatinine (mg/dL)	0.89 (0.44)	1.03 (0.43)	<0.001
BUN (mg/dL)	14.49 (5.42)	17.62 (8.53)	<0.001
Uric Acid (mg/dL)	5.47 (1.42)	5.88 (1.63)	<0.001
Albumin (g/dL)	4.27 (0.31)	4.18 (0.29)	<0.001
WBC (×10^9^/L)	7.00 (2.56)	7.57 (2.13)	<0.001
Hemoglobin (g/dL)	14.40 (1.45)	14.15 (1.50)	<0.001
Platelets (×10^9^/L)	261.34 (67.21)	255.07 (75.50)	0.005
MCV (fL)	90.79 (5.38)	91.26 (5.44)	0.085
Lymphocytes (×10^9^/L)	2.10 (1.74)	2.10 (0.87)	0.950

**Multivariable Logistic Regression Analysis:** Higher CTI levels were consistently associated with greater odds of PAD. Treated as a continuous variable, each unit increase in CTI was linked to higher PAD odds in the unadjusted model (OR 1.48, 95% CI 1.33-1.65, p < 0.001), the partially adjusted model (OR 1.55, 95% CI 1.37-1.76, p < 0.001), and the fully adjusted model (OR 1.47, 95% CI 1.25-1.75, p < 0.001) (Table 2). When CTI was evaluated in quartiles, higher odds of PAD were observed across increasing quartiles in unadjusted analyses (Table 3). Compared with Q1, unadjusted odds increased for Q2 (OR 1.65, 95% CI 1.19-2.30, p = 0.003), Q3 (OR 1.74, 95% CI 1.26-2.42, p < 0.001), and Q4 (OR 2.78, 95% CI 2.06-3.80, p < 0.001). After adjustment, the association was primarily driven by the highest quartile, with fully adjusted odds of 1.26 for Q2 (95% CI 0.88-1.80, p = 0.205), 1.34 for Q3 (95% CI 0.93-1.95, p = 0.114), and 2.07 for Q4 (95% CI 1.41-3.06, p < 0.001), while Q2 and Q3 did not reach statistical significance.

**Table 2:** **Association of Continuous CTI With PAD.***Odds ratios and 95% confidence intervals for the association between CTI (*per *unit) and peripheral artery disease across unadjusted, partially adjusted, and fully adjusted logistic regression models.*

Exposure	Unadjusted OR (95% CI)	Unadjusted p	Partially adjusted OR (95% CI)	Partially adjusted p	Fully adjusted OR (95% CI)	Fully adjusted p
CTI (per unit)	1.48 (1.33-1.65)	<0.001	1.55 (1.37-1.76)	<0.001	1.47 (1.25-1.75)	<0.001

**Table 3:** **Association of CTI Quartiles With PAD.***Odds ratios and 95% confidence intervals comparing CTI quartiles with Q1 as the reference group across unadjusted, partially adjusted, and fully adjusted logistic regression models.*

Quartile	Unadjusted OR (95% CI)	Partially adjusted OR (95% CI)	Fully adjusted OR (95% CI)	Unadjusted p	Partially adjusted p	Fully adjusted p
Q2 vs Q1	1.65 (1.19-2.30)	1.40 (1.00-1.99)	1.26 (0.88-1.80)	0.003	0.054	0.205
Q3 vs Q1	1.74 (1.26-2.42)	1.57 (1.11-2.22)	1.34 (0.93-1.95)	<0.001	0.011	0.114
Q4 vs Q1	2.78 (2.06-3.80)	2.67 (1.92-3.75)	2.07 (1.41-3.06)	<0.001	<0.001	<0.001

Survey-weighted analyses included 2744 participants, representing an estimated 42.8 million U.S. adults, with approximately 2.03 million weighted PAD events. Baseline characteristics were similar between included and excluded participants (Supplemental Table 1), with no statistically significant differences observed. In survey-weighted fully adjusted models, higher CTI levels remained significantly associated with greater odds of PAD. When modeled continuously, each unit increase in CTI was associated with higher odds of PAD (OR 1.77, 95% CI 1.28-2.44, p = 0.001) (Supplemental Table 2). When evaluated in quartiles, the association persisted across increasing CTI categories. Compared with Q1, fully adjusted odds were higher for Q2 (OR 2.14, 95% CI 1.14-4.00, p = 0.020), Q3 (OR 1.97, 95% CI 1.00-3.89, p = 0.049), and Q4 (OR 3.13, 95% CI 1.40-6.98, p = 0.008), with the highest CTI quartile demonstrating the greatest adjusted odds of PAD (Supplemental Table 3). Corresponding unadjusted and partially adjusted survey-weighted estimates are also presented in Supplemental Tables 2 and 3

**Fig. 2:**
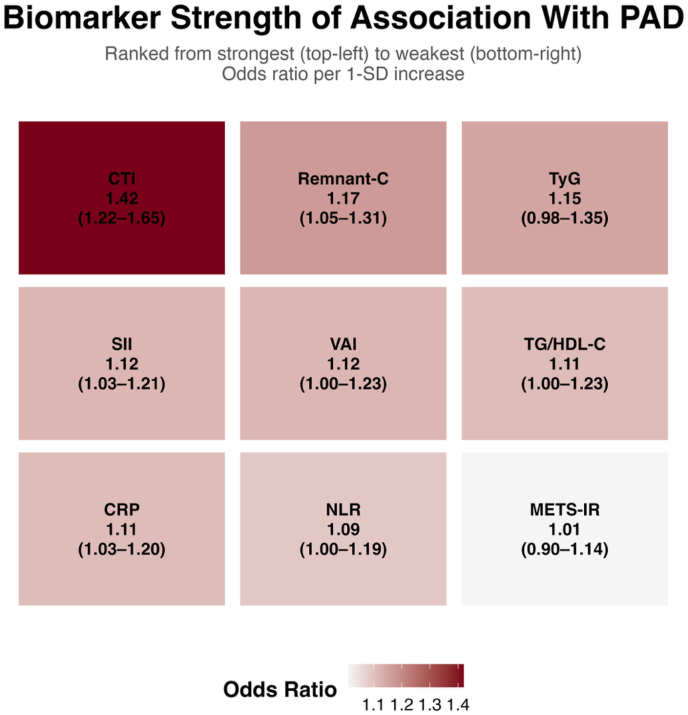
**Comparative Strength of Association Between Cardiometabolic Biomarkers and Peripheral Artery Disease.***Heatmap illustrating the relative strength of association between selected metabolic and inflammatory biomarkers and peripheral artery disease (PAD). Odds ratios and 95% confidence intervals are shown* per *1-standard deviation increase for each biomarker. Biomarkers are ranked from strongest (top left) to weakest (bottom right) based on effect size. CTI demonstrated the largest magnitude of association with PAD among the evaluated biomarkers, while other indices showed more modest associations.*

To provide broader context, we evaluated additional metabolic and inflammatory biomarkers in the full unweighted sample. When standardized to a 1-standard deviation increase, CTI demonstrated the strongest association with PAD among the evaluated biomarkers (Fig. 2). Other indices, including remnant cholesterol, TyG, SII, VAI, triglyceride-to-HDL cholesterol ratio, CRP, NLR, and METS-IR, showed comparatively smaller effect sizes, with some demonstrating weaker or borderline associations.

**Multinomial Logistic Regression Analysis:** CTI was also associated with lower ABI categories in multinomial analysis (Supplemental Table 4). Relative to adults with normal ABI, higher CTI levels were linked to greater odds of having borderline ABI of 0.90 to 0.99 (Relative Risk Ratio (RRR) 1.21, 95% CI 1.06-1.38, p = 0.005). The association was stronger for ABI less than 0.90, consistent with clinical PAD, where CTI showed a relative risk ratio of 1.52 (95% CI 1.28-1.80, p < 0.001).

**Restricted Cubic Spline Model:** The restricted cubic spline model suggested a positive association between CTI and the odds of PAD (Supplemental Fig. 1). The overall test for CTI was statistically significant (Wald chi-square 20.67, p = 0.0001), indicating that higher CTI levels suggest a greater PAD risk. However, the test for nonlinearity was not significant (Wald chi-square 0.78, p = 0.6781), suggesting that the relationship was predominantly linear across the observed CTI range. The spline curve demonstrated a steadily rising odds ratio with increasing CTI, with a sharper rise at higher values, while confidence intervals widened at the upper tail.

**Fig. 3:**
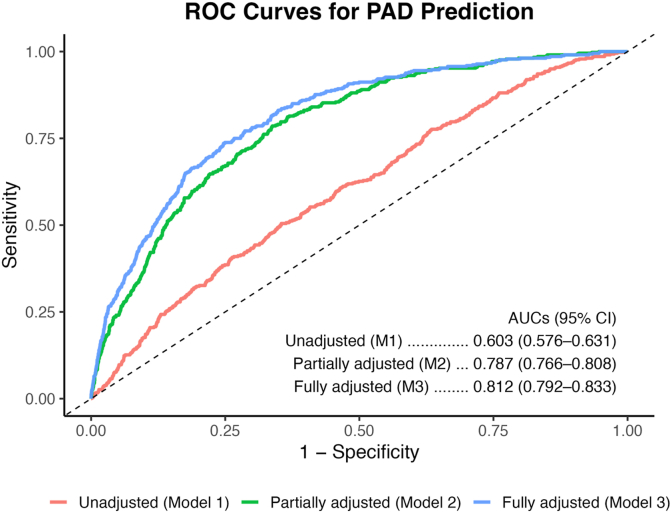
**ROC Curves for PAD Prediction Across CTI Models.** Receiver operating characteristic curves comparing the predictive performance of three CTI models for peripheral artery disease.

**Model Discrimination:** Model discrimination improved steadily with additional clinical adjustment (Fig. 3). The unadjusted CTI model had an AUC of 0.603 (95% CI 0.576-0.631). Adding demographic and metabolic covariates in the partially adjusted model increased the AUC to 0.787 (95% CI 0.766-0.808). The fully adjusted model reached an AUC of 0.812 (95% CI 0.792-0.833). In unadjusted analyses, CTI showed greater discriminative ability for PAD compared with the TyG index, with a higher AUC (0.603 vs 0.566), corresponding to a ΔAUC of 0.038 (p < 0.001). Consistent with this finding, CTI also showed improved model fit relative to TyG, as reflected by a lower AIC (ΔAIC = 28.6). Overall, CTI was associated with modest improvement in PAD discrimination, particularly when combined with standard clinical factors.

Likelihood ratio testing demonstrated that the addition of CTI significantly improved model fit beyond the fully adjusted base model (χ^2^ = 20.45, p < 0.001). Importantly, CTI also provided incremental value when added to models already containing comparator biomarkers, including base + TyG (χ^2^ = 22.21, p < 0.001), base + CRP (χ^2^ = 13.90, p < 0.001), and base + triglyceride-to-HDL cholesterol ratio (χ^2^ = 20.35, p < 0.001). These findings support that CTI contributes independent information beyond established cardiometabolic risk markers.

**Fig. 4:**
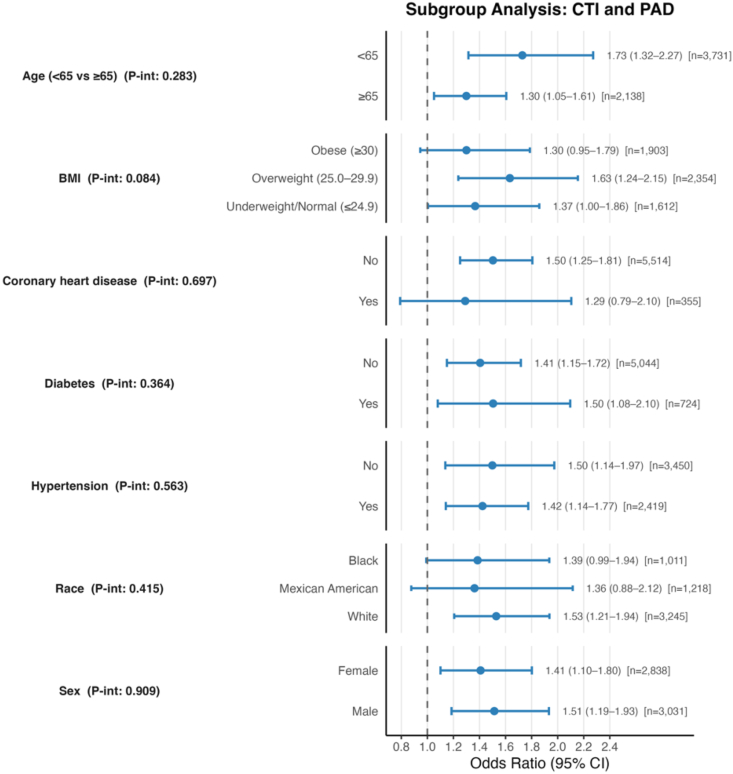
**Subgroup Analysis of the Association Between CTI and PAD.***Fully adjusted odds ratios with 95% confidence intervals for the association between continuous CTI and peripheral artery disease across key demographic and clinical subgroups. Interaction p-values are shown for each subgroup.*

**Subgroup Analysis & Interaction Test:** The association between CTI and PAD was consistent across all subgroups, and none of the interaction tests suggested meaningful heterogeneity (Fig. 4). Age did not modify the relationship (P-int = 0.283), and similar results were seen across BMI categories (P-int = 0.084). The association remained stable regardless of coronary heart disease (P-int = 0.697), diabetes (P-int = 0.364), or hypertension status (P-int = 0.563). Race and sex also showed no evidence of interaction, with P-int values of 0.415 and 0.909, respectively. Overall, no subgroup materially altered the strength or direction of the CTI-PAD association.

## 4 Discussion

Our study builds upon current work linking CTI with atherosclerotic disease driven by the synergistic combination of inflammation and metabolic dysfunction [19,20,23]. At a time when PAD remains underdiagnosed and undertreated despite advances in vascular imaging and medical therapy, there is a growing need for readily useable biomarkers that may aid in identifying disease. To our knowledge, this is the first study to demonstrate an association between CTI and PAD. When CTI was modeled as a continuous variable, each unit increase in CTI was associated with a 47% increase in PAD odds in the fully adjusted analyses, supporting a consistent association across the cohort. Quartile analyses demonstrated that the association was primarily driven by the highest CTI quartile, with individuals in the highest CTI quartile showing more than twice the odds of PAD compared with those in the lowest quartile. These findings indicate that CTI is associated with vascular risk across the cohort, although the quartile-based association was primarily driven by the highest quartile, consistent with a threshold effect at higher levels of metabolic-inflammatory burden. Importantly, these associations were consistent and of greater magnitude in nationally representative, survey-weighted analyses, supporting the consistency of the observed association across analyses at the population level. Together, these findings point to CTI as a complementary biomarker that may provide additional information within existing frameworks for vascular risk stratification by integrating routinely available laboratory markers.

As interest grows in refining the diagnostic and prognostic evaluation of PAD, multiple classes of inflammatory and metabolic biomarkers have been studied to complement traditional vascular assessment. High-sensitivity CRP (hs-CRP) is the most extensively validated inflammatory biomarker, with elevated levels conferring a relative risk of 1.86 for MACE and 3.49 for mortality in patients with PAD [24]. The American College of Cardiology (ACC) recommends universal screening with levels ≥3 mg/L corresponding to higher CV risk [25]. However, these thresholds were derived largely from secondary prevention populations and clinical trial cohorts enriched for high baseline inflammation. In contrast, our NHANES cohort demonstrated median baseline CRP values below 1 mg/L, yet CTI remained strongly associated with PAD. These findings highlight that integrating metabolic stress may help identify additional vascular risk even in individuals without overt systemic inflammation. Importantly, CRP alone is a well-established biomarker in PAD. Although CTI incorporates CRP as a component, it remains unclear whether the composite index provides meaningful incremental value beyond CRP itself. In this study, CTI demonstrated a stronger association with PAD than CRP in standardized analyses, although these comparisons are observational and should be interpreted cautiously. Notably, because CTI incorporates the natural logarithm of CRP, reduced sensitivity of standard CRP at lower levels may attenuate its ability to capture low-grade inflammation and contribute to more conservative effect estimates.

There is increasing recognition that inflammation is central to PAD, paralleling its established role in coronary artery disease. Landmark trials in coronary populations, including JUPITER, CANTOS, COLCOT, and LoDoCo2, established inflammation as both a major risk pathway and a modifiable therapeutic target, with consistent reductions in cardiovascular events independent of or complementary to lipid lowering (Supplemental Table 5) [[25], [26], [27]]. In contrast, while the role of inflammation in PAD is now documented, therapeutic data remains limited. No randomized trials have specifically tested anti-inflammatory therapies in PAD populations. A 2025 ACC Scientific Statement on inflammation and CVD recognized this gap and revealed that work on specialized pro-resolving mediator (SPM) pathways in PAD is still in early stages, with the OMEGA-PAD II trial showing increased SPM levels with fish oil but not yet demonstrating clinical benefit (Supplemental Table 5) [25].

Metabolic biomarkers further enrich the risk prediction framework in PAD by capturing physiologic pathways not reflected by inflammatory measures. Consistent with this framework, a 2025 ACC Scientific Statement on the management of PAD in adults with diabetes emphasizes metabolic risk assessment in PAD, noting that glycemic burden, dyslipidemia, and insulin resistance contribute to adverse limb and CV outcomes in addition to inflammatory pathways [28]. Triglycerides and fasting plasma glucose both demonstrate significant prognostic value in PAD. Higher levels of these markers are consistently linked to increased CV events, disease severity, and mortality risk [[29], [30], [31]]. Triglycerides ≥150 mg/dL independently predict adverse PAD outcomes, including a 37% higher rate of peripheral revascularization [29]. In patients with diabetes, elevated triglycerides correlate with greater PAD prevalence and severity, where adjusting for triglycerides reduces excess PAD risk attributed to diabetes (OR from 1.64 to 1.26) [32,33]. Insulin resistance in the form of elevated fasting plasma glucose or HbA1c predicts both incident PAD and CV mortality, where high fasting plasma glucose is now the leading global risk factor for PAD-related deaths and disability [30,31]. However, integrating these metabolic markers, the triglyceride-glucose (TyG) index has been associated with improved predictive performance, with each 1-SD increase raising incident PAD risk by 11.9% [34]. High TyG levels were associated with 42% increased odds of PAD, which was stronger in patients with diabetes [35].

As outlined above, numerous inflammatory and metabolic biomarkers have been individually linked to PAD, including the severity of the disease. However, each reflects only a single facet of a highly complex disease process. Studies such as Amrock and colleagues’ demonstrate that multi-marker strategies markedly improve risk stratification, and the enhanced prognostic performance of integrated metabolic indices like TyG further reinforce the value of combining biologic domains rather than relying on isolated markers [36]. Importantly, PAD remains underdiagnosed partly due to current screening tools relying on late-stage hemodynamic changes and isolated biomarker thresholds that fail to capture early disease biology. CTI may help address this gap by integrating metabolic dysfunction and inflammation into a single continuous metric derived from routinely obtained laboratory values. Unlike traditional approaches that depend on absolute cutoffs, CTI reflects coordinated biologic dysregulation, potentially enabling improved identification of PAD risk even in individuals who do not meet conventional inflammatory or metabolic thresholds. Within this context, CTI integrates multiple pathways and may better align with the multifactorial pathophysiology of PAD by merging inflammation and metabolic dysfunction. As the field becomes increasingly saturated with single-purpose biomarkers, composite indices like CTI may provide a potentially useful tool for prognostication of PAD. CTI may represent a scalable approach, although its clinical utility requires further validation. In addition, when evaluated alongside a broader set of metabolic and inflammatory biomarkers, CTI demonstrated the strongest association with PAD in standardized analyses, although these comparisons are observational and require further validation. While improvements in discrimination were modest, likelihood-based comparisons demonstrated that CTI provides statistically significant incremental information beyond both a fully adjusted clinical model and models incorporating established biomarkers.

Limitations of our study must be considered when interpreting the results. Although NHANES represents a large segment of the U.S. population, it excludes key groups such as individuals in nursing homes, incarcerated populations, and those in military settings. The dataset spans from 1999 to 2004, and advances in the diagnosis and management of PAD since then cannot be captured, which limits generalizability to modern patients. Importantly, these survey cycles were selected because they represent the most recent NHANES years in which ABI measurements were systematically obtained, enabling population-level assessment of PAD, as ABI data are not available in more recent NHANES cycles. ABI was not obtained as part of a population-wide screening protocol in NHANES and was measured only during the study examination, which may have resulted in incomplete identification of PAD and missed asymptomatic cases. ABI was measured at a single time point, raising the possibility of misclassification. Given that CTI and ABI were measured concurrently, this analysis is limited in its ability to establish temporality or causality, emphasizing the need for prospective studies to clarify temporal relationships. NHANES measured standard CRP rather than hs-CRP, which may underestimate low-grde inflammation, particularly at CRP levels below 1 mg/L. Because CTI incorporates the natural logarithm of CRP as a weighted component, reduced sensitivity in the lower CRP range may compress variability in CTI values and attenuate its ability to distinguish individuals with subtle inflammatory activation. As a result, the contribution of inflammation to the CTI signal may be diminished in this cohort, potentially leading to conservative effect estimates. In addition, threshold values derived from CTI in this analysis may not directly translate to settings where hs-CRP is used, as greater sensitivity at lower CRP levels could shift CTI distributions and risk stratification boundaries. Accordingly, the observed association between CTI and PAD may underestimate the true contribution of inflammation to vascular risk. Physical activity data are limited in NHANES, contributing to potential residual confounding. Laboratory values used to calculate CTI, including fasting glucose, triglycerides, and CRP, were measured once, restricting our ability to evaluate temporal changes. In addition, several comorbidities and lifestyle variables are self-reported, introducing the possibility of both under- and over-reporting.

## 5 Conclusion

To our knowledge, this is the first study to link CTI with PAD, extending its relevance beyond metabolic and coronary outcomes. These results broaden CTI's potential role in peripheral vascular disease and suggest that CTI may provide complementary information for cardiometabolic risk stratification and cardiovascular prevention. Additional prospective cohort studies are needed to determine whether CTI predicts incident PAD and improves early detection. Future studies should evaluate CTI in longitudinal cohorts with baseline biomarker assessment and serial vascular evaluation, including ABI measurements, to assess its ability to predict incident PAD, disease progression, and adverse cardiovascular and limb outcomes. Integration of CTI into risk prediction models alongside established clinical variables will be important to determine its incremental value for clinical screening and risk stratification.

## Disclosures

All authors have no conflicts of interest.

## Funding

None.

## Declaration of competing interest

The authors declare that they have no known competing financial interests or personal relationships that could have appeared to influence the work reported in this paper.
