# Supplementary material for: Association of the C-reactive protein-triglyceride-glucose index (CTI) with lower-extremity peripheral artery disease among U.S. adults
Source: Atheroscler Plus. 2026 May 19;65:100568. doi: 10.1016/j.athplu.2026.100568 (PMC13235333; doi:10.1016/j.athplu.2026.100568)
Supplement: Multimedia component 1 [file mmc1.pdf]

Supplement

Baseline Characteristics of Participants Excluded From and Included In the Survey-Weighted Analysis

Comparison performed within the unweighted analytic cohort

Variable	Excluded from survey-weighted analysis	Included in survey-weighted analysis	p-value
Age (years)	59.29 (13.05)	59.77 (12.78)	0.123
Sex			0.840
Male	51.5	51.8	
Female	48.5	48.2	
Race/Ethnicity			0.550
NH White	55.0	55.6	
NH Black	18.0	16.5	
Other Race	2.9	2.9	
Mexican American	20.2	21.3	
Other Hispanic	3.9	3.7	
Marital Status			0.376
Not Married	36.6	35.5	
Married	63.4	64.5	
Education Level			0.717
< High School	31.1	31.8	
High School/GED	23.9	23.0	
Some College	24.6	25.4	
College or Higher	20.4	19.8	
Income-to-Poverty Ratio	2.82 (1.61)	2.86 (1.61)	0.317
Hypertension			0.954
Yes	41.2	41.3	
Physical Activity			0.175
Yes	58.0	56.2	
No	42.0	43.8	
Coronary Heart Disease			0.952
Yes	6.1	6.0	
Heart Failure			0.538
Yes	3.2	3.5	
Diabetes			0.371
Yes	13.0	12.1	
Smoking			0.084
Yes	52.1	54.4	
BMI (kg/m²)	28.41 (5.55)	28.42 (5.46)	0.926
Hemoglobin A1c (%)	5.78 (1.14)	5.75 (1.09)	0.944
Total Cholesterol (mg/dL)	210.03 (42.17)	208.76 (41.18)	0.138
HDL (mg/dL)	52.98 (16.36)	52.47 (16.14)	0.215
Serum Creatinine (mg/dL)	0.90 (0.44)	0.89 (0.45)	0.152
Peripheral Artery Disease			0.683
Yes	7.0	7.3	

Supplemental Table 1. Baseline characteristics of participants included in and excluded from the survey-weighted analysis. *Baseline characteristics of participants included (n = 2,744) vs. excluded (n = 3,125) from survey-weighted analyses within the unweighted cohort (n = 5,869). Values are mean (SD) or %. Groups were similar across variables.*

Association of CTI with PAD — Continuous (Survey-weighted)

Odds ratios (95% CI) and *p* values across models

Exposure	Unadjusted OR (95% CI)	Unadjusted p	Partially adjusted OR (95% CI)	Partially adjusted p	Fully adjusted OR (95% CI)	Fully adjusted p
CTI (per unit)	1.65 (1.40–1.95)	<0.001	1.62 (1.30–2.04)	<0.001	1.77 (1.28–2.44)	0.001

Supplemental Table 2. Survey-Weighted Association of Continuous CTI With PAD. *Odds ratios (95% CI) for the association between CTI (per unit) and peripheral artery disease from survey-weighted logistic regression models using NHANES fasting weights across unadjusted, partially adjusted, and fully adjusted models.*

Association of CTI with PAD — Quartiles (ref = Q1) (Survey-weighted)

Odds ratios (95% CI) and *p* values across models

Quartile	Unadjusted OR (95% CI)	Partially adjusted OR (95% CI)	Fully adjusted OR (95% CI)	Unadjusted p	Partially adjusted p	Fully adjusted p
Q2 vs Q1	2.96 (1.64–5.33)	2.43 (1.32–4.45)	2.14 (1.14–4.00)	<0.001	0.005	0.020
Q3 vs Q1	2.90 (1.56–5.38)	2.31 (1.22–4.38)	1.97 (1.00–3.89)	0.001	0.012	0.049
Q4 vs Q1	4.62 (2.45–8.69)	3.75 (1.85–7.62)	3.13 (1.40–6.98)	<0.001	<0.001	0.008

Supplemental Table 3. Survey-Weighted Association of CTI Quartiles With PAD. *Odds ratios (95% CI) comparing CTI quartiles with Q1 as the reference group for peripheral artery disease from survey-weighted logistic regression models using NHANES fasting weights across unadjusted, partially adjusted, and fully adjusted models.*

Association of CTI with ABI Categories (Multinomial)

Relative Risk Ratios (95% CI) and *p* values; reference = Normal ABI (1.00–1.39)

Outcome contrast	CTI RRR (95% CI)	p-value
Borderline (0.90–0.99) vs Normal (1.00–1.39)	1.21 (1.06–1.38)	0.005
PAD (<0.90) vs Normal (1.00–1.39)	1.52 (1.28–1.80)	<0.001

Supplemental Table 4. Association of Continuous CTI With ABI Categories in Fully Adjusted Multinomial Models. *Relative risk ratios and 95% confidence intervals for the association between CTI (per unit) and ABI categories (0.90-0.99 and <0.90) compared with normal ABI (1.00-1.39) in fully adjusted multinomial logistic models*

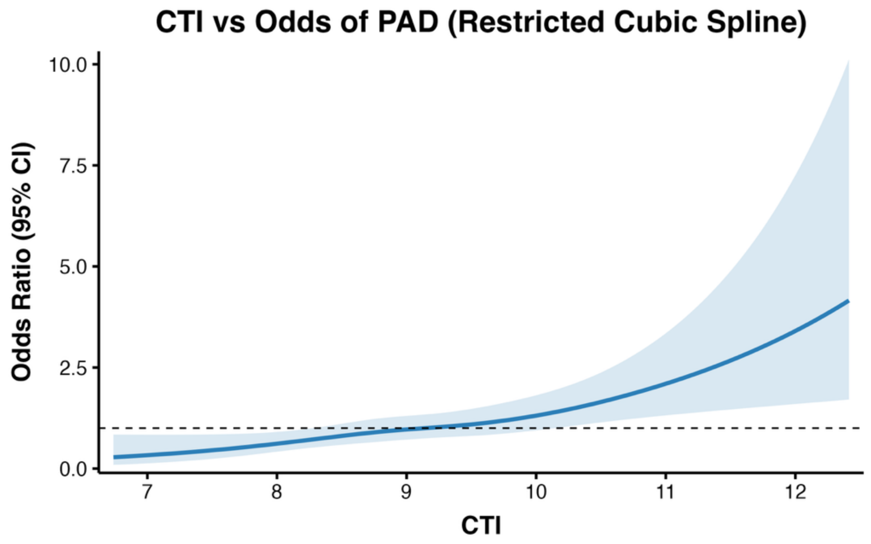

Supplemental Figure 1. Association of CTI With Odds of PAD Using Restricted Cubic Splines. *Restricted cubic spline analysis showing the adjusted association between continuous CTI values and the odds of PAD. The solid line represents the estimated odds ratio relative to the reference value, and the shaded region shows the 95% confidence interval. The spline model demonstrated a significant overall association between CTI and PAD, while the nonlinearity test was not significant, indicating a primarily linear relationship across the CTI range.*

Trial	Study Population	Intervention	Primary Inflammatory / Metabolic Target	Cardiovascular Outcomes
JUPITER	healthy men ≥50 years and women ≥60 years without prior cardiovascular disease, LDL-C <130 mg/dL, and high-sensitivity CRP ≥2.0 mg/L (n = 17,802)	Rosuvastatin 20 mg daily vs placebo	Systemic inflammation (hsCRP reduction) with concomitant lipid lowering	44% relative risk reduction in the primary composite CV endpoint (HR 0.56, 95% CI 0.46-0.69, p<.001); significant reductions in MI, stroke, arterial revascularization, and all-cause mortality
CANTOS	Patients with prior myocardial infarction and persistent residual inflammatory risk defined by hsCRP ≥2.0 mg/L despite optimal secondary prevention (n = 10,061)	Canakinumab (IL-1β monoclonal antibody) 50 mg, 150 mg, or 300 mg subcutaneously every 3 months vs placebo	IL-1β-mediated innate immune activation with downstream IL-6 and hsCRP reduction, without lipid lowering	Canakinumab 150 mg reduced the primary composite of nonfatal MI, nonfatal stroke, or CV death by 15% vs placebo (HR 0.85, 95% CI 0.74-0.98, p=0.021); significant reductions in recurrent MI and urgent revascularization; no effect on LDL cholesterol
COLCOT	Adults with recent myocardial infarction (within 30 days), receiving guideline-directed secondary prevention including statins and antiplatelet therapy (n = 4,745)	Colchicine 0.5 mg once daily vs placebo	Inhibition of innate immune activation via microtubule disruption and inflammasome modulation, with downstream reduction in inflammatory signaling	Colchicine reduced the primary composite endpoint of CV death, resuscitated cardiac arrest, MI, stroke, or urgent hospitalization for angina requiring revascularization (HR 0.77, 95% CI 0.61-0.96, p=0.02); largest reductions observed for stroke and urgent revascularization
LoDoCo2	Patients with chronic coronary disease, clinically stable for ≥6 months, receiving contemporary guideline-directed secondary prevention (n = 5,522)	Colchicine 0.5 mg once daily vs placebo	Broad suppression of innate immune activation through inhibition of microtubule polymerization and leukocyte-mediated inflammatory signaling	Colchicine reduced the primary composite endpoint of CV death, spontaneous MI, ischemic stroke, or ischemia-driven coronary revascularization (HR 0.69, 95% CI 0.57-0.83, p<0.01); significant reductions were also observed for key secondary composite endpoints
OMEGA-PAD II	Patients ≥50 years with symptomatic PAD and intermittent claudication (Rutherford I-III), ABI <0.9 or imaging-confirmed PAD (n = 24)	High-dose n-3 PUFA (fish oil) 4.4 g/day vs placebo for 3 months	Inflammation resolution pathways via increased omega-3 index and specialized pro-resolving lipid mediators (SPMs)	No significant changes in hsCRP, IL-6, or ICAM-1 were observed, but n-3 PUFA therapy increased the omega-3 index and multiple specialized pro-resolving lipid mediators, with nonsignificant trends toward improved claudication

Supplemental Table 4. Landmark Anti-Inflammatory and Metabolic Trials and Cardiovascular Outcomes. *Summary of major randomized clinical trials evaluating anti-inflammatory and metabolic interventions, their targeted inflammatory pathways, and associated cardiovascular outcomes across primary and secondary prevention populations.*
